# In Vitro and In Vivo Assessments of Two Newly Isolated Bacteriophages against an ST13 Urinary Tract Infection *Klebsiella pneumoniae*

**DOI:** 10.3390/v14051079

**Published:** 2022-05-17

**Authors:** Fanny Laforêt, Céline Antoine, Bob Blasdel Reuter, Johann Detilleux, Jean-Paul Pirnay, Sylvain Brisse, Abdoulaye Fall, Jean-Noël Duprez, Véronique Delcenserie, Damien Thiry

**Affiliations:** 1 Bacteriology Laboratory, Department of Infectious and Parasitic Diseases, FARAH and Faculty of Veterinary Medicine, University of Liège, 4000 Liège, Belgium; fanny.laforet@uliege.be (F.L.); celine.antoine@uliege.be (C.A.); jean-noel.duprez@uliege.be (J.-N.D.); 2 Food Science Department, FARAH and Faculty of Veterinary Medicine, University of Liège, 4000 Liège, Belgium; veronique.delcenserie@uliege.be; 3 Vésale Bioscience, Vésale Pharmaceutica, 5310 Noville-sur-Mehaigne, Belgium; bob.blasdel@phage.health; 4 Department of Animal Production-Quantitative Genetics, FARAH and Faculty of Veterinary Medicine, University of Liège, 4000 Liège, Belgium; jdetilleux@uliege.be; 5 Laboratory for Molecular and Cellular Technology (LabMCT), Queen Astrid Military Hospital, 1120 Brussels, Belgium; jean-paul.pirnay@mil.be; 6 Biodiversity and Epidemiology of Bacterial Pathogens, Institut Pasteur, Université Paris Cité, 75015 Paris, France; sylvain.brisse@pasteur.fr; 7 Genalyse Partner SA, En Hayeneux 62, 4040 Herstal, Belgium; afa@genalyse.eu

**Keywords:** bacteriophage, phage therapy, *Klebsiella pneumoniae*, ST13, K3, urinary tract infection, *Galleria mellonella*

## Abstract

Antibiotic resistance represents a major public health concern requiring new alternatives including phage therapy. *Klebsiella pneumoniae* belongs to the ESKAPE bacteria and can cause urinary tract infections (UTIs). The aims of this study were to isolate and characterize new bacteriophages against a *K. pneumoniae* strain isolated from UTIs and to assess their efficacy in vitro and in vivo in a *Galleria (G.) mellonella* larvae model. For this purpose, two bacteriophages were newly isolated against an ST13 *K. pneumoniae* strain isolated from a UTI and identified as K3 capsular types by *wzi* gene PCR. Genomic analysis showed that these bacteriophages, named vB_KpnP_K3-ULINTkp1 and vB_KpnP_K3-ULINTkp2, belong to the *Drulisvirus* genus. Bacteriophage vB_KpnP_K3-ULINTkp1 had the narrowest host spectrum (targeting only K3), while vB_KpnP_K3-ULINTkp2 also infected other *Klebsiella* types. Short adsorption times and latent periods were observed for both bacteriophages. In vivo experiments showed their ability to replicate in *G. mellonella* larvae and to decrease host bacterial titers. Moreover, both bacteriophages improved the survival of the infected larvae. In conclusion, these two bacteriophages had different in vitro properties and showed in vivo efficacy in a *G. mellonella* model with a better efficiency for vB_KpnP_K3-ULINTkp2.

## 1. Introduction

*Klebsiella pneumoniae* can roughly be classified into two pathotypes: the opportunistic ones, which are often multidrug-resistant (mdrKp), and the hypervirulent ones (hvKp). Today, drug-resistant properties coexist with hypervirulent ones [1], and this bacterium represents a huge public health concern. Indeed, “33,000 deaths per year in the EU and €1.5 billion per year in healthcare costs and productivity losses” [2] are figures related to antimicrobial resistance and *K. pneumoniae*, a member of the ESKAPE pathogens (*Enterococcus faecium, Staphylococcus aureus, Acinetobacter baumannii, Klebsiella pneumoniae, Pseudomonas aeruginosa,* and the *Enterobacter* species) [3], is on the WHO’s “critical priority” list of antibiotic-resistant bacteria to guide the discovery of new control methods [4]. This bacterium, naturally present in the human gastrointestinal and respiratory tracts, belongs to the *Enterobacteriaceae* family. At least 78 different capsular serotypes, K-types, of *K. pneumoniae* were identified at the beginning of the 20th century [5]. However, a century later, some of them were newly identified as *K. oxytoca*, *K. variicola*, *K. quasipneumoniae*, *K. michiganensis*, *Raoutella ornithinolytica*, *R. planticola*, and *R. terrigena* [6,7,8,9]. This pathogen is responsible for many infections such as pneumonia, sepsis, liver abscesses, and urinary tract infections (UTIs). Although most UTIs are caused by uropathogenic *Escherichia coli* (UPEC), *K. pneumoniae* is considered the second most frequent pathogenic bacterium [10], especially in diabetic patients [11,12]. According to the current Belgian recommendations, first-choice treatments against UTIs include nitrofurantoin or fosfomycin therapies [13]. However, the in vitro sensitivity of *K. pneumoniae* against these antimicrobials decreases, especially in extended-spectrum-beta-lactamase bacteria (ESBL) [14]. New treatment alternatives are urgently needed, and phage therapy represents a promising approach. Bacteriophages are the most abundant biological entities in the world and are responsible for maintaining the balance in bacterial populations owing to their capacity to selectively infect and kill bacterial hosts [15]. As such, bacteriophages live in different biological systems where bacteria are present, such as the urinary tract [16,17]. As therapeutic agents against UTIs, bacteriophages have already proven their value, especially against UPEC but also against *K. pneumoniae* infections [18,19,20]. The use of bacteriophages against *K. pneumoniae* has already been studied in other contexts than UTIs, with promising results [21]. Indeed, in a murine model of pneumonia caused by *K. pneumoniae*, treatment with the bacteriophage reduced the number of bacteria in the lungs as well as neutrophil and lymphocyte infiltrations [22]. In another in vivo model, a *Galleria (G.) mellonella* larvae model, the mortality rate of the infected larvae with an ESBL-producing mdrKp decreased with bacteriophage injection (pre- and post-bacterial infection) [23]. The same model was used to test the therapeutic effects of bacteriophages on survival of larvae infected with ST258 or ST23 *K. pneumoniae* and on a multiple bacterial infection with *K. pneumoniae, E. coli*, and *E. cloacae* (with repeated treatment injections) [24,25].

The aims of this study were to isolate and characterize new bacteriophages against a *K. pneumoniae* strain isolated from UTIs and to assess their efficacy in vitro and in vivo in a *(G.) mellonella* larvae model. 

## 2. Materials and Methods

### 2.1. Bacterial Strain 

The bacterial strain selected for the bacteriophages’ isolation, *K. pneumoniae* QAMH 130326/0185, was previously isolated from a urine sample of a Belgian diabetic patient with a UTI. Its capsular type (K-type, K) was identified by *wzi* gene PCR [5], and the phenotypic antibiotic susceptibility profile of this strain was assessed by disk diffusion assay using Mueller-Hinton plates (Becton Dickinson, Erembodegem, Belgium), Axonlab disks (Belgium), and the SIRscan micro software (Axonlab, Machelen, Belgium). The determination of the colistin minimum inhibitory concentration (MIC) was performed by broth dilution assay in FRCOL 96-well microtiter plates, according to the manufacturer’s instructions (Sensititre, Fisher Scientific, Merelbeke, Belgium) [26]. The results were interpreted using EUCAST (European Committee on Antimicrobial Susceptibility Testing) clinical breakpoints (2021 edition) and CASFM VET (Comité de l’Antibiograme de la Société Française de Microbiologie) standards (2019 edition).

To analyze the genome of this bacterium, bacterial genomic DNA was obtained using the DNeasy Blood & Tissue kit (Qiagen, Crawley, UK), following the manufacturer’s recommendations. Sequencing libraries were constructed using Nextera XT Illumina kit (Illumina, San Diego, CA, USA). The libraries were then sequenced on a Miseq system paired-end 2 × 300 bp read length (Illumina, San Diego, CA, USA). Contig assembly and scaffolding were performed using SPAdes Assembler V3.10 [27]. A first automatic genome annotation was performed using RAST server [28]. Virulence factor search and pathogenic typing were performed using the Kleborate tool (https://github.com/katholt/Kleborate, accessed on 23 February 2022) [29] available on the Pathogenwatch platform (https://pathogen.watch, accessed on 21 February 2022) [30] and Resfinder (https://cge.cbs.dtu.dk/services/ResFinder, accessed on 3 March 2022) [31].

### 2.2. Bacteriophage Isolation 

In order to isolate new bacteriophages, 5 mL of the supernatants of centrifuged (10 min at 9500× *g*) and ultrafiltered (0.2 µm) wastewaters were brought into contact with the *K. pneumoniae* QAMH 130326/0185 (100 µL at optical density (OD) (600 nm) between 0.2 and 0.3) in 5 mL of twice concentrated Luria-Bertani (LB) broth (Sigma-Aldrich, Saint-Louis, MO, USA) enriched with MgSO_4_ (1 mM) and CaCl_2_ (1 mM). This solution, as well as a sterility control of the wastewater and a bacterial growth control, were incubated at 37 °C for 4 h until bacterial lysis. The lysate and the bacterial growth broths were centrifuged (9500× *g* for 10 min) and filtered (0.2 μm) and were spread (4 µL) on enriched LB agar with a *K. pneumoniae* QAMH 130326/0185 bacterial overlay technique. After incubation at 37 °C for 4 h, one individual plaque was selected and purified four times following the same procedure. Transmission electronic microscopy (TEM) pictures were obtained on purified bacteriophages using a Tecnai Spirit microscope (FEI, Eindhoven, The Netherlands) operating at 120 kV at the electron microscopy unit of Sciensano (Bruxelles, Belgium). The bacteriophages were purified by CsCl density gradient (layers of 1.33, 1.45, 1.50, and 1.70 g/cm^3^) ultracentrifugation (133,900× *g*; 3 h; 4 °C), dialysis using Slide-A-Lyzer di-alysis cassettes G2 (Thermo Fisher Scientific Inc., Merelbeke, Belgium). Four µL of both bacteriophages were plated on a *K. pneumoniae* QAMH130326/0185 overlay to analyse the plaque of lysis morphologies.

### 2.3. Bacteriophages Genome Sequencing 

To analyse the genomes of these bacteriophages, genomic DNA was extracted from CsCl purified bacteriophages using the DNeasy Blood & Tissue kit (Qiagen, Crawley, UK). The sequencing libraries were obtained using the Illumina Nextera XT DNA sample preparation kit and sequenced using a MiSeq Illumina Next instrument (Illumina, San Diego, CA, USA). The bacteriophage genomes were assembled into single contigs using SPAdes Assembler V3.10 [27]. The web server RAST was used for genome annotation with coding DNA sequences (CDS) outputs. These results were then compared with the web server HHpred (https://toolkit.tuebingen.mpg.de/tools/hhpred, accessed on 1 February 2022) [32]. Homologous related bacteriophages were found in GenBank using BLASTn analysis (https://blast.ncbi.nlm.nih.gov/Blast.cgi?PAGE_TYPE=BlastSearch, accessed on 1 February 2022), and closely related bacteriophage genomes were illustrated with Easyfig [33]. The automated prediction of the cycle of both bacteriophages was assessed with PhageAI [34].

### 2.4. Temperatures and pH Stability

A total of 1 mL of both bacteriophages, at 10^8^ plaque-forming units (PFU)/mL, was incubated for 1 h at different temperatures (25 °C, 37 °C, 45 °C, and 60 °C), in biological triplicate, before titration. The pH stability was also assessed in biological triplicate: 100 µL of both bacteriophages, at 10^8^ PFU/mL, were ten times diluted in PBS at different pH (2, 4, 6, 8, 10, and 12) and then titrated. 

After the investigation of the normality of each distribution (corresponding to the titration data for each phage and for both incubation condition) (by a histogram, a quantile-quantile plot (QQ-plot), a boxplot, and a Shapiro-Wilk test), a one-way analysis of variance (ANOVA) (with multiple comparisons) was assessed to highlight if the concentration obtained after 1 h incubation was significant different from the original. All statistical analyses were performed with R (*p*-value ≤ 0.05) [35].

### 2.5. Host Range and Efficiency of Plating

The host range of both bacteriophages was assessed using 31 different *Klebsiella* and 8 UPEC *E. coli* strains (Table S1). Each bacteriophage was spotted (4 µL) in duplicate on the different bacterial overlays (OD: 0.15–0.25) and incubated at 37 °C until the appearance of lysis plaques. The results were divided in three classes: no lysis, confluent lysis or individual lysis plaques. Then, if there was lysis (confluent lysis or just individual plaques), the efficiency of plating (EOP, the ratio between the concentration of the tested strain over the concentration of the propagation strain) was determined (Table S1). To that end, each bacteriophage was tenfold serially diluted in PBS (in triplicate), drops (2 µL) were plated on enriched LB agar with the different strains overlayers, and Petri dishes were then incubated for 4 h at 37 °C. 

### 2.6. Adsorption Times and Low MOI Kinetic Curves

The adsorption times and the low multiplicity of infection (MOI) kinetic curves (MOI of 0.1) were determined in biological triplicate. For the adsorption time, the bacteriophage was mixed with the host bacterium (QAMH 130326/0185) at MOI of 0.1 and incubated for 10 min at 37 °C. Every 2 min, 100 µL of the solution was sampled and 10 times diluted in PBS to reduce the interaction of the bacteriophage with the bacteria. After 10 min, all samples were filtrated (0.2 µm), and the unadsorbed bacteriophages were titrated to measure the adsorption time [36]. The adsorption rate constants were calculated according to the following mathematical formula:

(1) $$k=\frac{2.3}{Bt}\log\frac{P0}{P}$$

where *k* is the adsorption rate constant, in mL/min; *B* is the concentration of bacterial cells; and *t* is the time interval in which the titre falls from *P*0 (the original concentration of the bacteriophage) to *P* (the final concentration of the bacteriophage) [37].

For the low MOI kinetic curves, a mix of bacteriophage and bacteria similar to that prepared for the adsorption times was performed in biological triplicate, incubated at 37 °C till the bacteriophage’s adsorption, and then centrifugated (13,000× *g,* 1 min). The pellet was resuspended in 10 mL of LB Lennox and incubated at 37 °C with shaking over 100 min. Every 5 min, 100 µL were sampled, 10 times diluted in PBS, filtrated (0.2 µm), and then titrated to highlight the latent periods and the steady state times.

### 2.7. In Vitro Bacteriophage Activity

A total of 100 μL of *K. pneumoniae* QAMH130326/0185 (at 10^6^ colony formCFU/mL) and different concentrations of each bacteriophage (1000 μL, 100 μL, and 10 μL at 10^8^ PFU/mL for MOIs 1000, 100, and 10) were mixed in enriched LB Lennox broth (5 mL) in biological triplicates. Three control tubes were added to the experiment: a growth control, a sterility control, and a bacteriophage control. All tubes were incubated at 37 °C with shaking, and optical densities (OD 600) were measured every hour for 12 h.

### 2.8. Survival Rate in Galleria mellonella Larvae

The optimal bacterial inoculation dose which can kill 80% of the larvae within 4 days was determined in one preliminary experiment (data not shown). Then, the assessment of the survival rates for infected larvae treated with the bacteriophages was performed in a single experiment for MOIs of 10 and 100. A total of 150 larvae were distributed in 5 groups (Table 1), and each larva was inoculated using an automatic injector (Cole Parmer, Vernon Hills, IL, USA) in the last left proleg for the first injection followed by a second injection 1 h later in the last right proleg. Injections of 10 µL were performed using BD Plastipak™ 1 mL sterile syringes (Becton-Dickinson, Franklin Lakes, NJ, USA) and sterile 30-gauge needles (Terumo corporation, Tokyo, Japan). The larvae were incubated at 37 °C, and their survival was evaluated every 24 h. Kaplan-Meier survival curves were generated to assess the survival of the different groups using Rcommander [27]. Log-rank tests were performed to highlight any significant difference in survival rates between the groups (*p*-value ≤ 0.05). Then, if the MOI of 100 was not sufficient to improve the survival rates of the insects, another single experiment with an MOI of 1000 was performed (Table 1). 

### 2.9. Bacterial Load and Bacteriophage Replication in Galleria mellonella Larvae

The second main in vivo experiment aimed to assess the bacterial and bacteriophage titre evolutions [36,38]. For this single experiment, 120 larvae were divided into 6 groups and inoculated following the same protocol (using an MOI of 100). Bacterial and bacteriophage titrations were performed after 24 h and 72 h in technical triplicate, with larvae ground in a homogenizer (Stomacher) in groups of 10 individuals. The mix (200 µL) was diluted in PBS and weighed. For the bacteriophage titre, half of the sample was filtered using a 0.2 µm sterile syringe filter (ref.514-0073, VWR, Leicestershire, UK), 10-fold serially diluted in PBS, plated (2 µL) on LB agar with a QAMH 130326/0185 overlay and incubated at 37 °C 4 h. For the bacterial titration, the diluted samples were plated on SCAi agar (Simmons Citrate Agar, Inositol 1% and ampicillin 2 µg/mL, Sigma-Aldrich, Saint-Louis, MO, USA) and incubated at room temperature for 24 h. 

After the investigation of the normality of each distribution (corresponding to the bacterial and phage titration data for each phage) (by a histogram, a quantile-quantile plot (QQ-plot), a boxplot, and a Shapiro-Wilk test), a two-way non-parametric ANOVA (with multiple comparisons) was assessed to highlight if there were significant differences between groups and during all the experiments. All the statistical analyses were performed with R (*p*-value ≤ 0.05) [35].

## 3. Results

### 3.1. Bacterial Strain 

The *K. pneumoniae* QAMH 130326/0185 isolate was characterized as KL3 capsular locus (KL) and K-type (K) K3 (*wzi*-40 allele). This strain showed resistance to amoxicillin + clavulanic acid, ampicillin, cefuroxime, and fosfomycin, as well as intermediate susceptibility to cefquinome and temocillin (Table S2). 

The genomic analysis assigned this 5,628,480 bp length bacteria to the sequence type (ST) ST13. Two virulence factors were found: the genotoxin colibactin *clb*-3 and the yersinibactin *ybt*-17, both included in integrative and conjugative element (ICE, ICE-*Kp10*. Regarding the antibiotic resistance elements, 3 acquired (bla_OXA-1_, *sul*-1 and *aadA1*) and 3 intrinsic (*fosA, oqxAB* and bla*SHV-1*) genes were found. At the protein level, proteins FimA, B, D, F, G, and H were predicted.

Sequencing was submitted as NCBI BioProject PRJNA821689. 

### 3.2. Bacteriophage Isolation 

**Figure 1:**
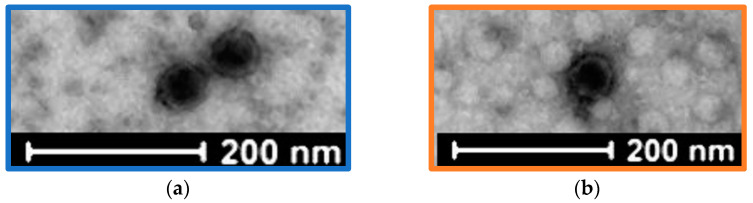
Negative staining transmission electron microscopic images: (**a**) vB_KpnP_K3-ULINTkp1 and (**b**) vB_KpnP_K3-ULINTkp2.

Two bacteriophages (vB_KpnP_K3-ULINTkp1 and vB_KpnP_K3-ULINTkp2) were isolated from wastewaters collected in Belgium in 2020 (Brussel (North Station) for vB_KpnP_K3-ULINTkp1 and Liège (Oupeye) for vB_KpnP_K3-ULINTkp2). The TEM pictures showed a *Podoviridae* morphology with a non-enveloped icosahedral head, a diameter of approximately 55 nm, and a very short tail for both bacteriophages (Figure 1). The lysis plaques showed halo zones and diameters of around 3 mm and 1 mm respectively for vB_KpnP_K3-ULINTkp1 and vB_KpnP_K3-ULINTkp2 (Figure S1).

### 3.3. Bacteriophage Genome Sequencing

**Figure 2:**
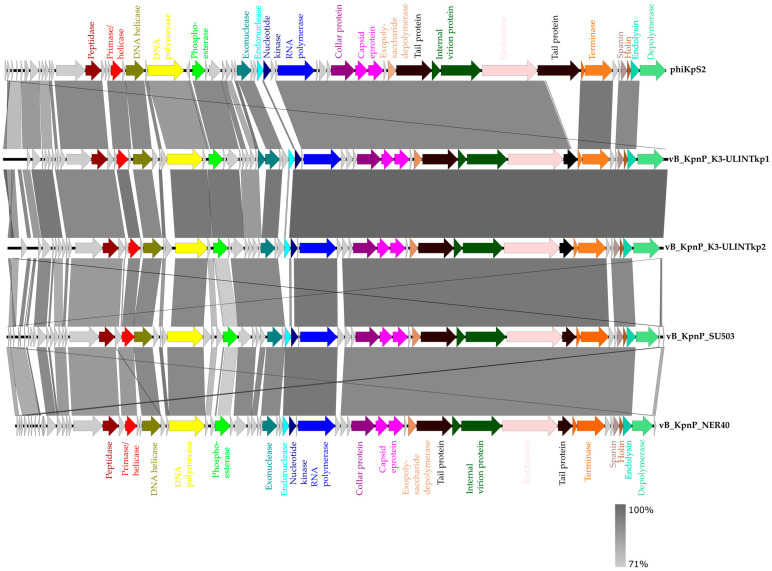
Comparative genomics of bacteriophages vB_KpnP_K3-ULINTkp1 and vB_KpnP_K3-ULINTkp2 with bacteriophages phiKpS2, vB_KpnP_SU503, and vB_KpnP_NER40.

According to the closely related bacteriophage genomes, both bacteriophages belong to the *Caudovirales* order, *Autographiviridae* family, *Slopekvirinae* subfamily, and *Drulisvirus* genus with lengths of 44,290 bp and 43,687 bp respectively for vB_KpnP_K3-ULINTkp1 and for vB_KpnP_K3-ULINTkp2. The bacteriophage vB_KpnP_K3-ULINTkp1 shared identity with the bacteriophages phiKpS2 (91.30%), vB_KpnP_NER40 (92.08%), and vB_KpnP_SU503 (94.34%) (Figure 1). The bacteriophage vB_KpnP_K3-ULINTkp2 shared identity with the bacteriophages phiKpS2 (91.33%), vB_KpnP_NER40 (92.21%), and vB_KpnP_SU503 (94.22%) (Figure 2). Both bacteriophage cycles were predicted as virulent (at 98.51% for vB_KpnP_K3-ULINTkp1 and 98.5% for vB_KpnP_K3-ULINTkp), and no lysogenic or virulent genes were revealed. In silico, both bacteriophages contained putative proteins such as depolymerases, endonucleases, and endolysins.

The sequences of the phages were submitted on the NCBI BioProject PRJNA821689.

### 3.4. Temperatures and pH Stability

**Figure 3:**
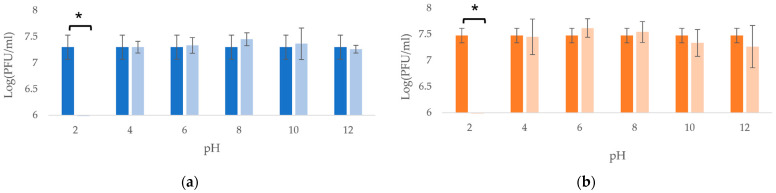
pH stability of the bacteriophages (**a**) vB_KpnP_K3-ULINTkp1 and (**b**) vB_KpnP_K3-ULINTkp2 after 1 h of pH treatment at 25 °C. The mean value of 3 titrations (±σ) is represented and the concentration measured before testing is represented with dark bars. *p*-value (*) ≤ 0.05.

**Figure 4:**
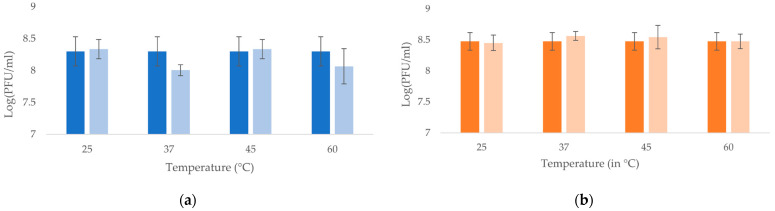
Temperature stability of the bacteriophages (**a**) vB_KpnP_K3-ULINTkp1 and (**b**) vB_KpnP_K3-ULINTkp2 after 1 h at the desired temperature (in °C). The means of 3 titrations (±σ) are represented, and the concentrations measured before testing are represented with dark bars.

Both bacteriophages showed stable lytic activity over the pH range of 4–12 (Figure 3), and both bacteriophages were thermostable until at least 60 °C (Figure 4).

### 3.5. Host Range and Efficiency of Plating

Thirty-nine strains were tested, chosen to represent a diversity of *Klebsiella* and *E. coli* UPEC. The vB_KpnP_K3-ULINTkp1 bacteriophage was able to lyse a ST490 KL107 K3 *K. pneumoniae* (SB5904) and a *K. oxytoca* strain (QAMH 121209/0028) with EOPs of 0.0072 and 0 (0 corresponds to lysis at host range but no plaques at EOP) (Table S1). The vB_KpnP_K3-ULINTkp2 bacteriophage was able to lyse a ST490 KL107 K3 *K. pneumoniae* (SB5904), a K81 *K. pneumoniae* (QAMH 130526/0682), a ST17 KL107 *K. pneumoniae* (SB5890), a *K. oxytoca* (QAMH 121209/0028), and a K30 *K. pneumoniae* (ATCC27736) and showed EOPs of 0.0455, 0.0001, 0.0009, 0.0007, and 0 respectively (Table S1).

### 3.6. Adsorption Times and Low MOI Kinetic Curves

**Figure 5:**
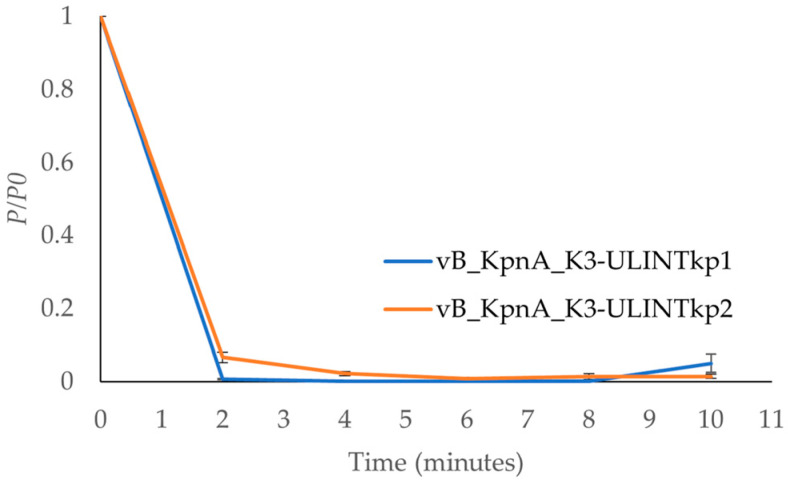
Adsorption time of the bacteriophages vB_KpnP_K3-ULINTkp1 (blue curve) and vB_KpnP_K3-ULINTkp2 (orange curve). *P*0, initial bacteriophage concentration; *P*, number of free bacteriophages.

**Figure 6:**
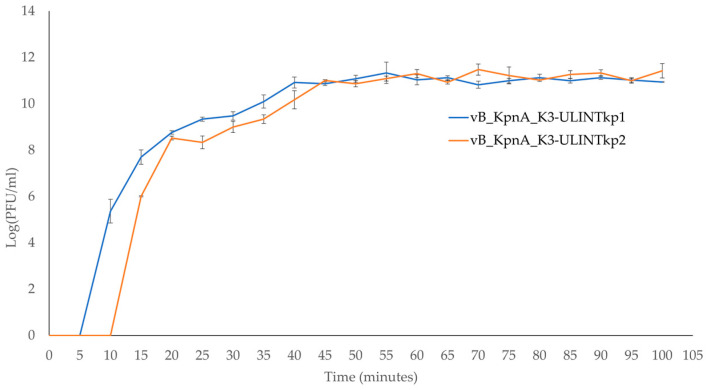
Low MOI bacteriophage kinetic curves of the bacteriophages vB_KpnP_K3-ULINTkp1 (blue curve) and vB_KpnP_K3-ULINTkp2 (orange curve).

The times needed for the bacteriophages’ adsorption were 4 min for vB_KpnP_K3-ULINTkp1 and 6 min for vB_KpnP_K3-ULINTkp2 (Figure 5), with *k* constants of 1.66 × 10^−8^ (mL/min) and 8.5 × 10^−9^ (mL/min) respectively. Regarding the latent periods, they lasted 5–10 min for vB_KpnP_K3-ULINTkp1 and 10–15 min for vB_KpnP_K3-ULINTkp2 and reached a steady state (around 10^11^ PFU/mL) after 40 min and 45 min respectively (Figure 6).

### 3.7. In Vitro Bacteriophage Activity

The in vitro efficacy assessment of the bacteriophages showed a lytic activity for both bacteriophages (irrespective of MOI used) (Figure S2).

### 3.8. Survival Rate in Galleria mellonella Larvae

**Figure 7:**
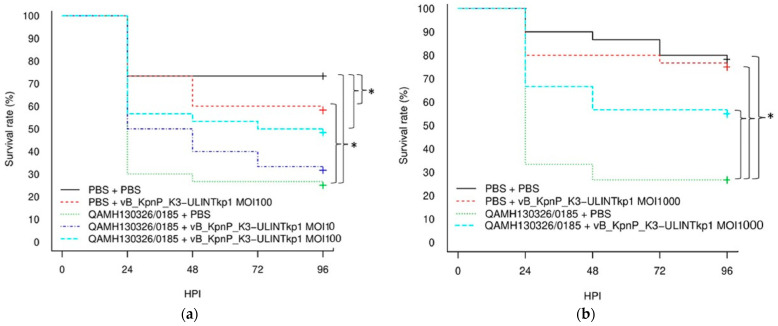

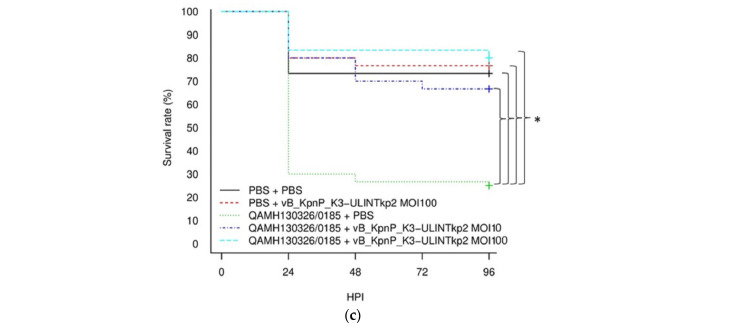
Kaplan–Meier survival curves of the experiments with *G. mellonella* larvae inoculated with *K. pneumoniae* QAMH130326/0185 or PBS and treated with vB_KpnP_K3-ULINTkp1 (**a**) for MOI = 10, MOI = 100 and (**b**) for MOI = 1000 or vB_KpnP_K3-ULINTkp2 (**c**) or PBS 1 h later. Each group contained 30 larvae. MOI: multiplicity of infection; HPI: hours post inoculation; *p*-value (*) ≤ 0.05 (Log-rank tests).

Compared with the infected but not treated group, the infected larvae treated with vB_KpnP_K3-ULINTkp1 showed a better survival rate only with an MOI of 1000 (53% vs. 27%), in contrast with vB_KpnP_K3-ULINTkp2, which already improved the survival at a MOI of 10 (67% vs. 23%) (Figure 7).

### 3.9. Bacterial Load and Bacteriophage Replication in Galleria mellonella Larvae

**Figure 8:**
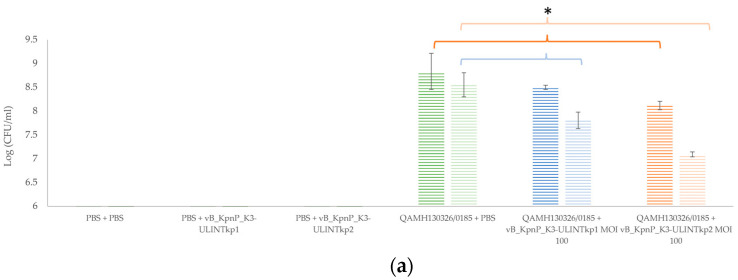

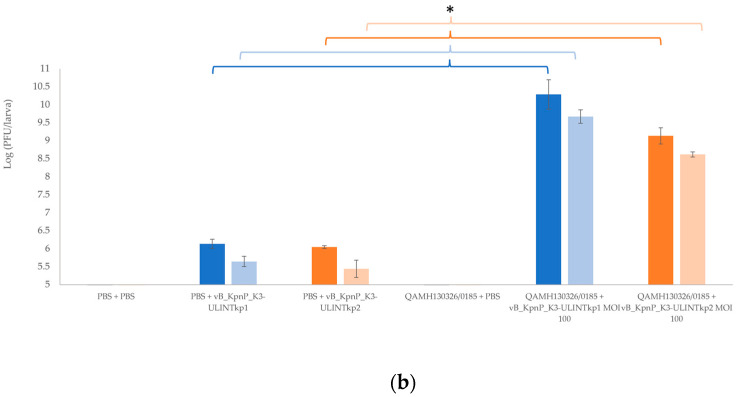
Bacterial (hatched chart) (**a**) and bacteriophage (**b**) titrations at 24 h (dark bars) and 72 h (light bars) after inoculation of the *G. mellonella* larvae in the titration experiments with the bacteriophages vB_KpnP_K3-ULINTkp1 or vB_KpnP_K3-ULINTkp2 and *K. pneumoniae* QAMH130326/0185. The mean of 3 titrations (±σ) is represented. The significant results including groups with a titration equal to 0 (PFU/larvae or CFU/larvae) are not shown. MOI: multiplicity of infection. *p*-value (*) ≤ 0.05.

A significantly higher bacteriophage concentration was observed for both bacteriophages in the groups infected and treated (2 × 10^10^ PFU/larvae for vB_KpnP_K3-ULINTkp1 and 10^9^ PFU/larvae for vB_KpnP_K3-ULINTkp2) compared with the bacteriophage control groups at 24 h (10^6^ PFU/larvae for both bacteriophages) (Figure 8). Similar significant results were observed at 72 h with concentrations of 5 × 10^9^ PFU/larvae for vB_KpnP_K3-ULINTkp1 and 4 × 10^8^ PFU/larvae for vB_KpnP_K3-ULINTkp2 in the infected-treated group instead of 4 × 10^5^ PFU/larvae for vB_KpnP_K3-ULINTkp1 and 3 × 10^5^ (PFU/larvae) for vB_KpnP_K3-ULINTkp2 in the bacteriophage control groups. Regarding the bacterial concentrations (Figure 8), after 24 or 72 h, significantly higher concentrations of bacteria was observed in the infected and untreated group compared with the group infected and treated with vB_KpnP_K3-ULINTkp2. For vB_KpnP_K3-ULINTkp1, a significant bacterial concentration decrease was observed only at 72 h. Indeed, the bacterial concentrations were around 7 × 10^8^ (CFU/larvae) and 4 × 10^8^ (CFU/larvae) for 24 h and 72 h respectively. For the vB_KpnP_K3-ULINTkp1 group, the concentrations varied from 3 × 10^8^ (CFU/larvae) at 24 h to 6 × 10^7^ (CFU/larvae) at 72 h, while they ranged from 10^8^ (CFU/larvae) to 10^7^ (CFU/larvae) for vB_KpnP_K3-ULINTkp2.

## 4. Discussion

The two bacteriophages isolated and characterized in this study showed predicted virulent cycles, a productive infection with short adsorptions and latent periods during the low MOI kinetic curve, and as such, they could represent an interesting alternative to antibiotics. Clear lysis plaques with a halo zone suggest that they possess an exopolysaccharide-degrading enzyme, highlighted in silico [39]. They were stable over large pH and temperature ranges, in accordance with activity in human urine, which exhibits a pH ranging from 5.4 to 7.2. The presence of endolysins has been observed in silico, these hydrolases digesting the rigid bacterial peptidoglycan wall leading to bacterial lysis and death. With a fast bacterial lysis, with a high specificity, with the possibility of molecular engineering to change the host range or allow to cross the outer membrane, with activity against biofilms, and with a low risk of resistance emergence, these so-called enzybiotics seem promising [40]. In addition, putative endonucleases were found. These enzymes are believed to reduce the horizontal gene transfer by transformation by avoiding the release of intact plasmid in the environment [41].

The K-type 3 (K3) clusters are observed only in *K. pneumoniae* species. Their capsules are rich in mannose residues [42] used for interactions with mannose receptors that are particularly present on macrophages’ surfaces [43]. K3 includes two subspecies: *Klebsiella pneumoniae* subsp. *pneumoniae* and *Klebsiella pneumoniae* subsp. *rhinoscleromatis*. The subspecies *pneumoniae* could be isolated from fresh urine or from stool of diarrheic patients [44]. The subspecies *rhinoscleromatis* is the agent of nasal and upper respiratory chronic infection, rhinoscleroma. The latter can be genetically differentiated from the other by MLST, by the absence of *kfu* (an iron uptake marker), and by the presence of *rmpA* (regulator of mucoid phenotype A) [45]. With the type 1 fimbrial adhesin, *K. pneumoniae* has the capacity to colonize the urinary tract (UT) as the putative protein FimA is responsible for bladder cell invasion and biofilm development [46,47], FimH can link the mannose-binding receptor of the UT [47], and FimD, F, and G are minor subunits also involved in UT invasion [48]. Although some ST13 *K. pneumoniae* are multidrug-resistant bacteria [49,50], this was not the case for the strain QAMH130326/0185 even when it was phenotypically resistant to fosfomycin, a first-line urinary antibiotic. 

*Klebsiella* bacteriophage specificity for the capsular type is well-known [25,51]. This specificity could be linked to a specific interaction of the bacteriophage’s depolymerase, highlighted in silico for both bacteriophages in this study, with the bacterial capsule [52,53,54], even if a broad host range of *K. pneumoniae* bacteriophages have already been described [24,55]. In this study, vB_KpnP_K3-ULINTkp1 seemed more specific than vB_KpnP_K3-ULINTkp2 and could replicate within two of the tested strains (QAMH 130326/0185 and SB5904) that both belonged to K-type 3. Conversely, vB_KpnP_K3-ULINTkp2 could replicate in four other K-type strains but with a low production EOP (between ≤0.1 and ≥0.001). The host range and the EOP showed that both bacteriophages were unable to replicate in some strains but still produced lytic spots at high concentrations. This phenomenon, reflecting a putative abortive infection, could represent a non-productive bacteriophage infection which kills the bacteria [56]. 

The bacteriophages phiKpS2, vB_KpnP_NER40, and vB_KpnP_SU503 were genomically related to these newly isolated bacteriophages. The first has been isolated with the *K. pneumoniae* DSM2026 mutant (*K. pneumoniae* S2) in an abnormal propanediol fermentation broth [57]. The second had been isolated in the Chermyankan river with a K2 *K. pneumoniae* (Kp40) and showed a narrow spectrum to this capsular type [58]. The third had been isolated using the *K. pneumoniae* strain 07RAFM-KPN-503, and could replicate, throughout the host range experiment, in another *K. pneumoniae* strain, 07RAFM-KPN-510 [59,60].

The behaviours of vB_KpnP_K3-ULINTkp1 and vB_KpnP_K3-ULINTkp2 differ in vivo. Indeed, the infected *G. mellonella* larvae treated with vB_KpnP_K3-ULINTkp1 showed a higher survival rate only at MOI of 1000 with 53% of survival instead of 27% in the infected and nontreated group. The treatment with the vB_KpnP_K3-ULINTkp2 bacteriophage seems to be more efficient, with 77% survival at MOI of 100 and 67% at MOI of 10 instead of 23% for the infected larvae. The bacterial titration experiments showed that both bacteriophages seemed to decrease the bacterial load compared with the infected group. However, the efficacy of vB_KpnP_K3-ULINTkp2 was better than that of vB_KpnP_K3-ULINTkp1 with a lower bacterial load at 24 h and 72 h. Both bacteriophages seemed to replicate in the larvae, with higher concentrations obtained in the treated group.

## 5. Conclusions

Two newly isolated *Klebsiella* bacteriophages demonstrated their efficacy in vitro and in vivo by increasing the survival of *G. mellonella* larvae. Even if this did not result in a complete elimination of the inoculated bacteria, these results suggest that the two studied bacteriophages could efficiently prevent a *K. pneumoniae* urinary tract infection induced by this ST13 KL3 K3 strain. Although these bacteriophages are genomically closely related, vB_KpnP_K3-ULINTkp2 showed better efficacy in the in vivo *G. mellonella* model and a broader host spectrum than vB_KpnP_K3-ULINTkp1. Both conditions being interesting, a narrow spectrum avoids a microbiota disruption in complex microbial systems such as the intestine, but a broad spectrum increases the success of treatment in less complex microbial systems such as the urinary tract, where *Klebsiella* is not usually found in healthy people.

## Figures and Tables

**Table 1:** Groups of *G. mellonella* larvae inoculated for the survival and titration experiments (MOIs of 10, 100 and 1000).

		1st Injection (10 µL)	2nd Injection (10 µL)
Experiment with MOI 100 and MOI 10	1	PBS	PBS
	2	QAMH130326/0185: 10^4^ (CFU)	PBS
	3	QAMH130326/0185: 10^4^ (CFU)	vB_KpnA_K3-ULINTkp1 (or kp2): 10^5^ (PFUl)
	4	QAMH130326/0185: 10^4^ (CFU)	vB_KpnA_K3-ULINTkp1 (or kp2): 10^6^ (PFU)
	5	PBS	vB_KpnA_K3-ULINTkp1 (or kp2): 10^6^ (PFU)
Experiment with MOI 1000	1	PBS	PBS
	2	QAMH130326/0185: 10^4^ (CFU)	PBS
	3	QAMH130326/0185: 10^4^ (CFU)	vB_KpnA_K3-ULINTkp1: 10^7^ (PFU/10 µL)
	4	PBS	vB_KpnA_K3-ULINTkp1: 10^7^ (PFU)

MOI: Multiplicity of Infection; CFU: Colony-Forming Units; PFU: Plaque-Forming Units.

## Data Availability

Sequencing was submitted as NCBI BioProject PRJNA821689.

## Supplementary Materials

The following supporting information can be downloaded at: https://www.mdpi.com/article/10.3390/v14051079/s1. Table S1, the host range and efficiency of plating (EOP) of vB_KpnA_K3-ULINTkp1 and vB_KpnA_K3-ULINTkp2 and the characteristics of the *Klebsiella* (**a**) and *E. coli* UPEC (**b**) strains used. Table S2, phenotypic antibiotic susceptibility profile of the strain *Klebsiella pneumoniae* QAMH130326/0185. Figure S1, pictures of the plaques of lysis of the bacteriophages (**a**) vB_KpnA_K3-ULINTkp1 and (**b**) vB_KpnA_K3-ULINTkp2. Figure S2, in vitro assessment, in biological triplicate, of the efficacy of the bacteriophages vB_KpnA_K3-ULINTkp1 (**a**) and vB_KpnA_K3-ULINTkp2 (**b**) against *K. pneumoniae* 130326/0185 with optical density, OD (600 nm).
