# Supplementary material for: In Vitro and In Vivo Assessments of Two Newly Isolated Bacteriophages against an ST13 Urinary Tract Infection Klebsiella pneumoniae
Source: Viruses. 2022 May 17;14(5):1079. doi: 10.3390/v14051079 (PMC9144312; doi:10.3390/v14051079)
Supplement: Supplementary file 1 [file viruses-14-01079-s001.zip › viruses-1684844-supplementary.pdf]

Table S1. Host range and efficiency of plating (EOP) of vB_KpnA_K3-ULINTkp1 and vB_KpnA_K3-ULINTkp2 and the characteristics of the *Klebsiella* **(a)** and *E. coli* UPEC **(b)** strains used.

(a)

Identification of the strains of *Klebsiella*									Host range & EOP	
**N°**	**Species**	***wzi* allele**	**K**	**K_ locus**	**O_locus**	**ST**	**CG/ SL**	**Strain**	**vB_KpnA_K3 -ULINTkp1**	**vB_KpnA_K3 -ULINTkp2**
**1**	*K. pneumoniae*		1			23		SB4385	x	x
**2**	*K. pneumoniae*	*wzi*-14	14					QAMH 121231/0018	x	x
**3**	*K. pneumoniae*	*wzi*-27	27					SHIME 2.1	x	x
**4**	*K. pneumoniae*	*wzi*-84	28					QAMH 121014/0096	x	x
**5**	*K. pneumoniae*	*wzi*-85	30					ATCC 27736	x	NP
**6**	*K. pneumoniae*	*wzi*-37	37	KL50	O1v1	35	11	SB5908	x	x
**7**	*K. pneumoniae*	*wzi*-59	3	KL107	OL3/ OL3a	490	8	SB5904	0,0072	0,0455
**8**	*K. pneumoniae*	*wzi*-81	81					QAMH 130528/0682	x	0,0001
**9**	*K. pneumoniae*	*wzi*-101	24	KL107	OL2v1	2444	9	SB5906	x	x
**10**	*K. pneumoniae*	*wzi*-141		KL107	OL5	494	7	SB5902	x	x
**11**	*K. pneumoniae*	*wzi*-141		KL107	OL5	17	3	SB5890	x	0,0009
**12**	*K. pneumoniae*	*wzi*-141		KL107	OL5	17	15	SB6038	x	x
**13**	*K. pneumoniae*	*wzi*-149	62	KL107	OL2v1	2552	10	SB5907	x	x
**14**	*K. pneumoniae*	*wzi*-235		KL107	OL1v1	107	14	SB6037	x	x
**15**	*K. pneumoniae*	*wzi*-253		KL107	OL1v2	3137	1	SB5885	x	x
**16**	*K. pneumoniae*	*wzi*-368		KL107	OL3/ OL3a	1426	16	SB6043	x	x
**17**	*K. pneumoniae*	*wzi*-466		KL107	OL3/ OL3a	3138	4	SB5891	x	x
**18**	*K. pneumoniae*			KL35	OL1v1	305	12	SB6070	x	x
**19**	*K. pneumoniae*			KL107	OL3/ OL3a	3139	13	SB6036	x	x
**20**	*K. pneumoniae*							QAMH 110628/0752	x	x
**21**	*K. pneumoniae*							QAMH 080708/0488-2	x	x
**22**	*K. pneumoniae*							NCTC 13438	x	x
**23**	*K. pneumoniae*			KL5	OL1v1	29	18	SB6063	x	x
**24**	*K. pneumoniae*			KL106		258		SB4551	x	x
**25**	*K. oxytoca*							QAMH 110607/1254	x	x

**26**	*K. oxytoca*							QAMH 121209/0028	NP	0,0007
**27**	*K. oxytoca*							QAMH 121120/0608	x	x
**28**	*K. variicola*	*wzi*-170		KL5	OL3/ OL3a	2594	6	SB5901	x	x
**29**	*K. variicola*	*wzi*-352		KL113	OL3/ OL3a	347	5	SB5897	x	x
**30**	*K. variicola*	*wzi*-115	54	KL113	OL1v1	29	18	SB6060	x	x
**31**	*K. quasi-pneumoniae*	*wzi*-537		KL107	OL12	705	2	SB5886	x	x

(b)

**Identification of the strains**							**Host range**	
**N°**	**Species**	***neuC* (K1)**	***kfib* (K5)**	***TEM***	**CTX-M**	**Strain**	**vB_KpnA_K3-ULINTkp1**	**vB_KpnA_K3-ULINTkp2**
**1**	*E. coli*				CTX-M 1	15	x	x
**2**	*E. coli*				CTX-M 15	142.1	x	x
**3**	*E. coli*	*neuC+*			CTX-M 15	143.1	x	x
**4**	*E. coli*				CTX-M 1	144.1	x	x
**5**	*E. coli*				CTX-M 1	144.2	x	x
**6**	*E. coli*		*kfib+*	TEM 1	CTX-M 1	151.1	x	x
**7**	*E. coli*				CTX-M 1	154.2	x	x
**8**	*E. coli*					J96	x	x

K. pneumoniae: *Klebsiella pneumoniae*; *K. oxytoca*: *Klebsiella oxytoca*; *K. variicola*: *Klebsiella variicola*: *K. quasipneumoniae*: *Klebsiella quasipneumoniae*; ST: sequence type; CG/SL: clonal group/sublineages; K: capsular type; K_locus: K antigen and O_locus: O antigen ; *E. coli*: *Escherichia coli*

Table S2. Phenotypic antibiotic susceptibility profile of the strain *Klebsiella pneumoniae* QAMH130326/0185.

**Method**	**Antibiotics**	**Results (in mm)**	**Interpretation**
	Amoxicilin + clavulanic acid	17	**R**
	Ampicilin	6	**R**
	Aztreonam	34	S
	Cefotaxim	31	S
	Cefotaxim + clavulanic acid	32	Not a ESBL
	Cefoxitin	25	S
	Cefuroxim	16	**R**
**Disk duffision**	Fosfomycine	16	**R**
	Gentamycine	24	S
	Meropenem	31	S
	Nitrofurantoin	19	S
	Norflaxacin	39	S
	Temocilin	21	**I**
	Trimethoprim + sulfamethoxazole	26	S
	Ceftiofur	28	S
	Cefquinon	21	**I**
**MIC**	Colistin	0,5	S

References EUCAST 2021 for: amoxicilin + clavulanic acid, ampicilin, aztreonam, cefotaxime, cefotaxime + clavulanique acid, cefoxitin, cefuroxim, fosfomycine, gentamycine, meropenem, nitrofurantoin, norflaxacin, temocilin, trimethoprim + sulfamethoxazole, and colistin. References CASFM VET 2019 for: ceftiofur and cefquinon. S: susceptible to the antibiotic; R: resistant to the antibiotic; I: susceptible at higher dosage to the antibiotic; MIC: minimal inhibitory concentration.

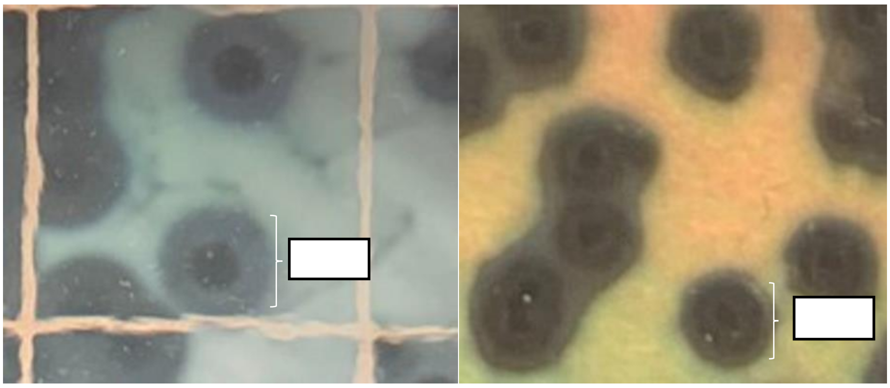

(a) (b)

Figure S1. Pictures of the plaques of lysis of the bacteriophages **(a)** vB_KpnA_K3-ULINTkp1 and **(b)** vB_KpnA_K3-ULINTkp2.

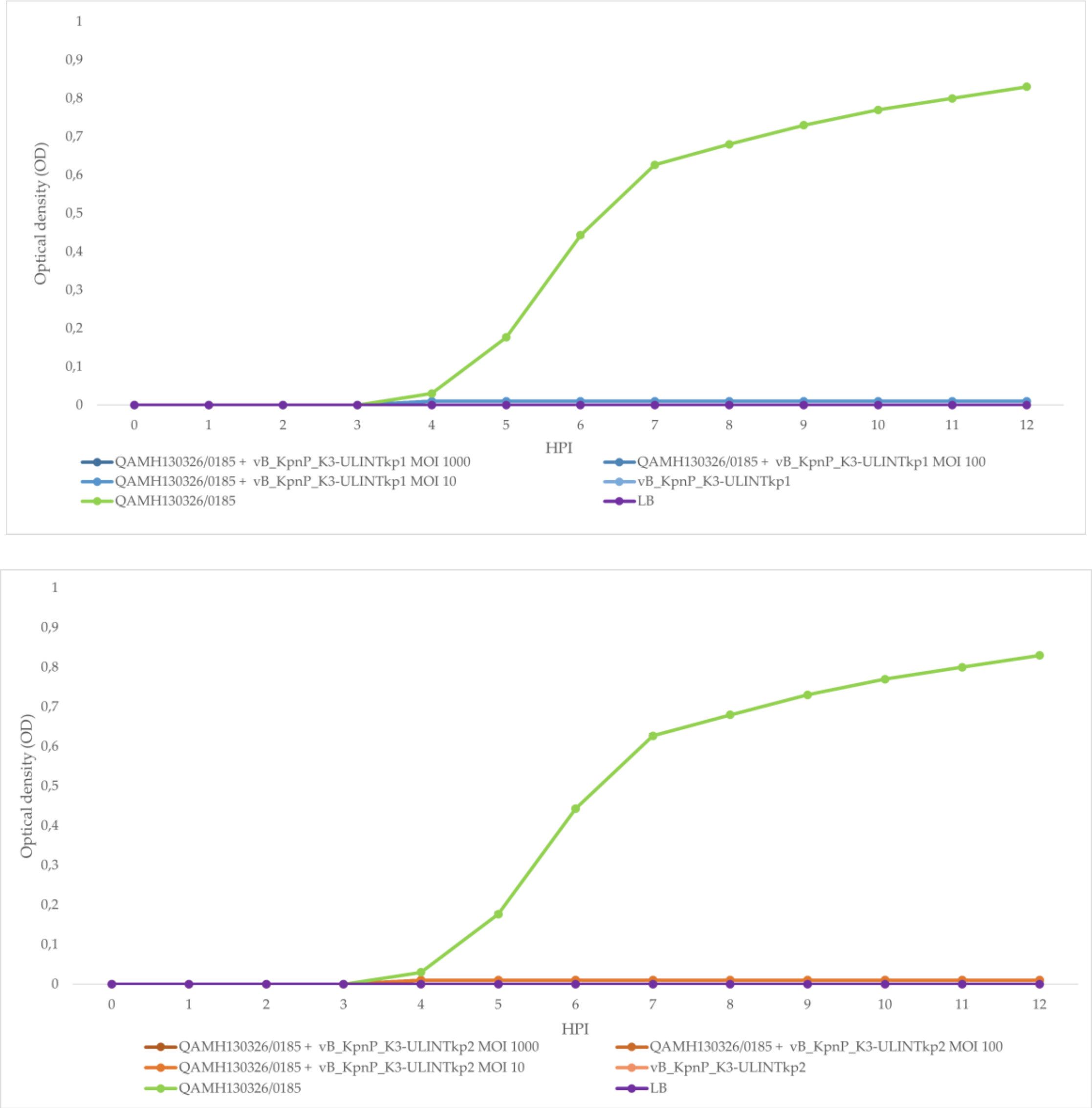

(a)

(b)

Figure S2. In vitro assessment, in biological triplicate, of the efficacy of the bacteriophages vB_KpnA_K3-ULINTkp1 **(a)** and vB_KpnA_K3-ULINTkp2 **(b)** against *K. pneumoniae* 130326/0185 with the measure of the optical density, OD (600nm). HPI: hours post inoculation.
